# Supplementary material for: Genome- and Transcriptome-Wide Characterization of bZIP Gene Family Identifies Potential Members Involved in Abiotic Stress Response and Anthocyanin Biosynthesis in Radish (Raphanus sativus L.)
Source: Int J Mol Sci. 2019 Dec 16;20(24):6334. doi: 10.3390/ijms20246334 (PMC6941039; doi:10.3390/ijms20246334)
Supplement: Supplementary file 1 [file ijms-20-06334-s001.zip › Figure S2.docx]

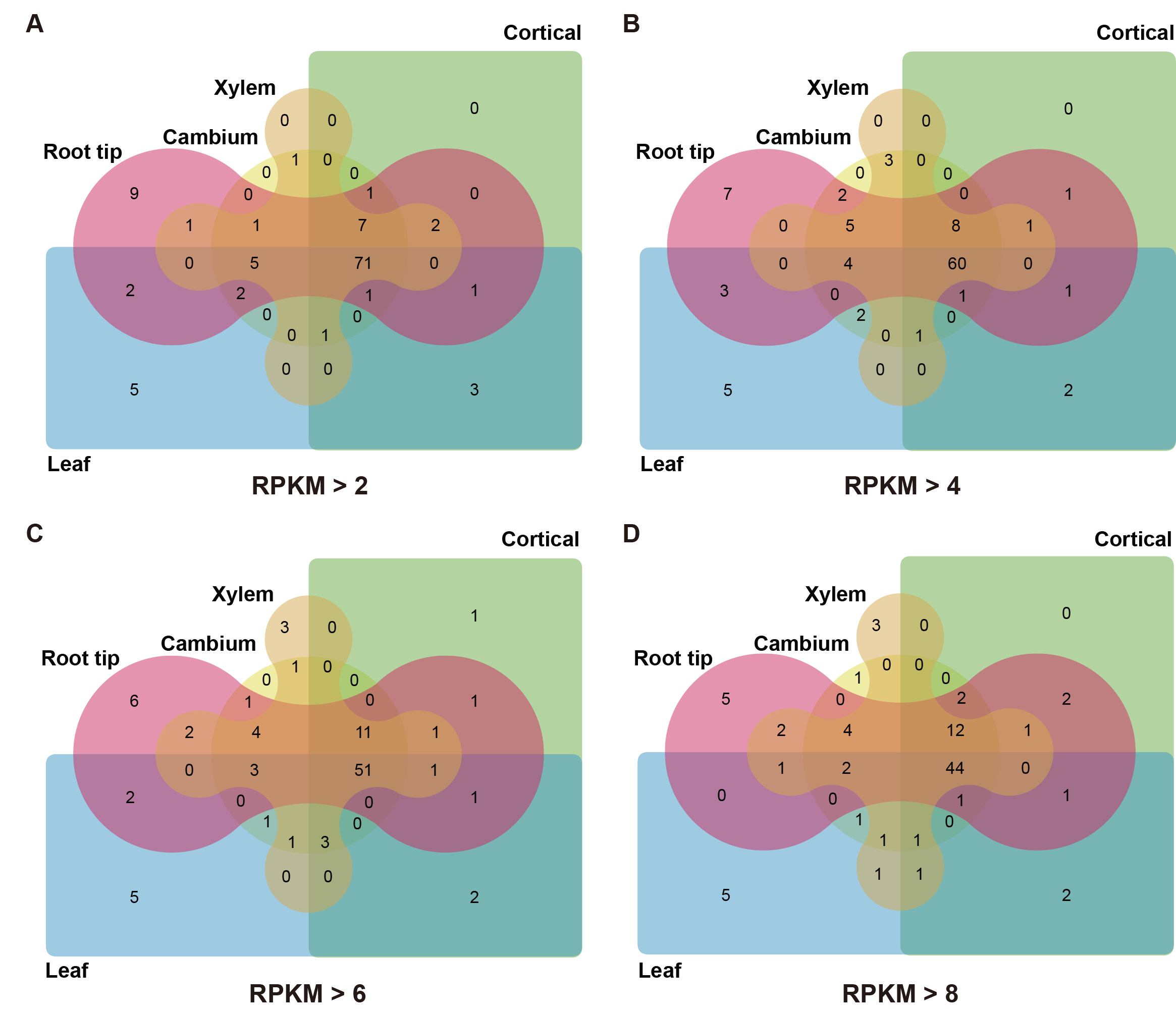


**Figure S2.** Venn diagrams of overlapping *RsbZIP*s that are expressed in leaf, cortical, cambium, xylem and root tip when RPKM are greater than 2, 4, 6 and 8, respectively
