# Supplementary material for: Genome- and Transcriptome-Wide Characterization of bZIP Gene Family Identifies Potential Members Involved in Abiotic Stress Response and Anthocyanin Biosynthesis in Radish (Raphanus sativus L.)
Source: Int J Mol Sci. 2019 Dec 16;20(24):6334. doi: 10.3390/ijms20246334 (PMC6941039; doi:10.3390/ijms20246334)
Supplement: Supplementary file 1 [file ijms-20-06334-s001.zip › Figure S3.docx]

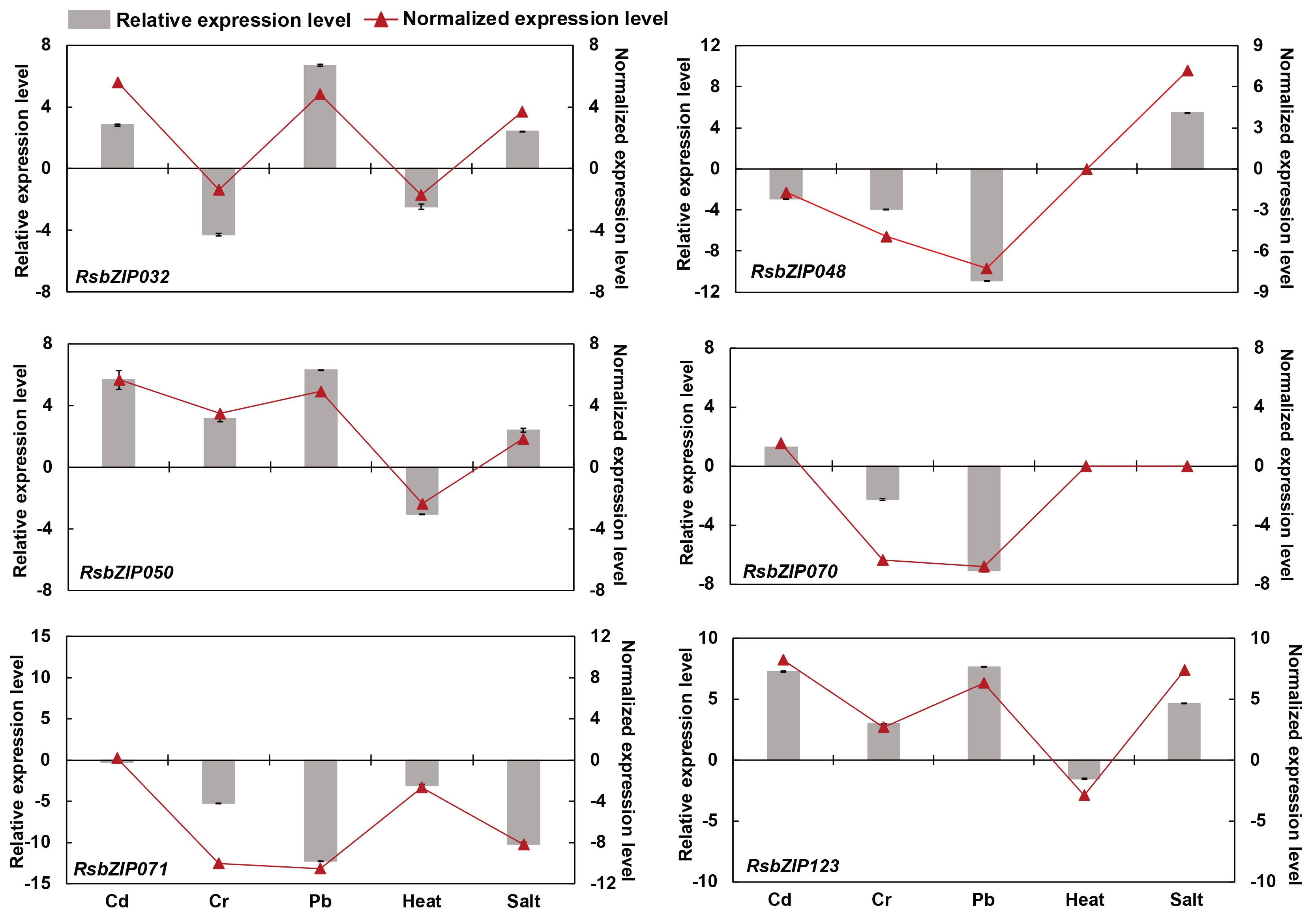


**Figure S3.** RT-qPCR validation of selected six *RsbZIP*s under Cd, Cr, Pb, heat and salt treatments in radish taproot. Red bar represents transcript abundance changes calculated by the RPKM method. Gray bar with associated standard error bar indicates relative expression level determined by RT-qPCR analysis
