# Supplementary material for: Genome- and Transcriptome-Wide Characterization of bZIP Gene Family Identifies Potential Members Involved in Abiotic Stress Response and Anthocyanin Biosynthesis in Radish (Raphanus sativus L.)
Source: Int J Mol Sci. 2019 Dec 16;20(24):6334. doi: 10.3390/ijms20246334 (PMC6941039; doi:10.3390/ijms20246334)
Supplement: Supplementary file 1 [file ijms-20-06334-s001.zip › Figure S4.docx]

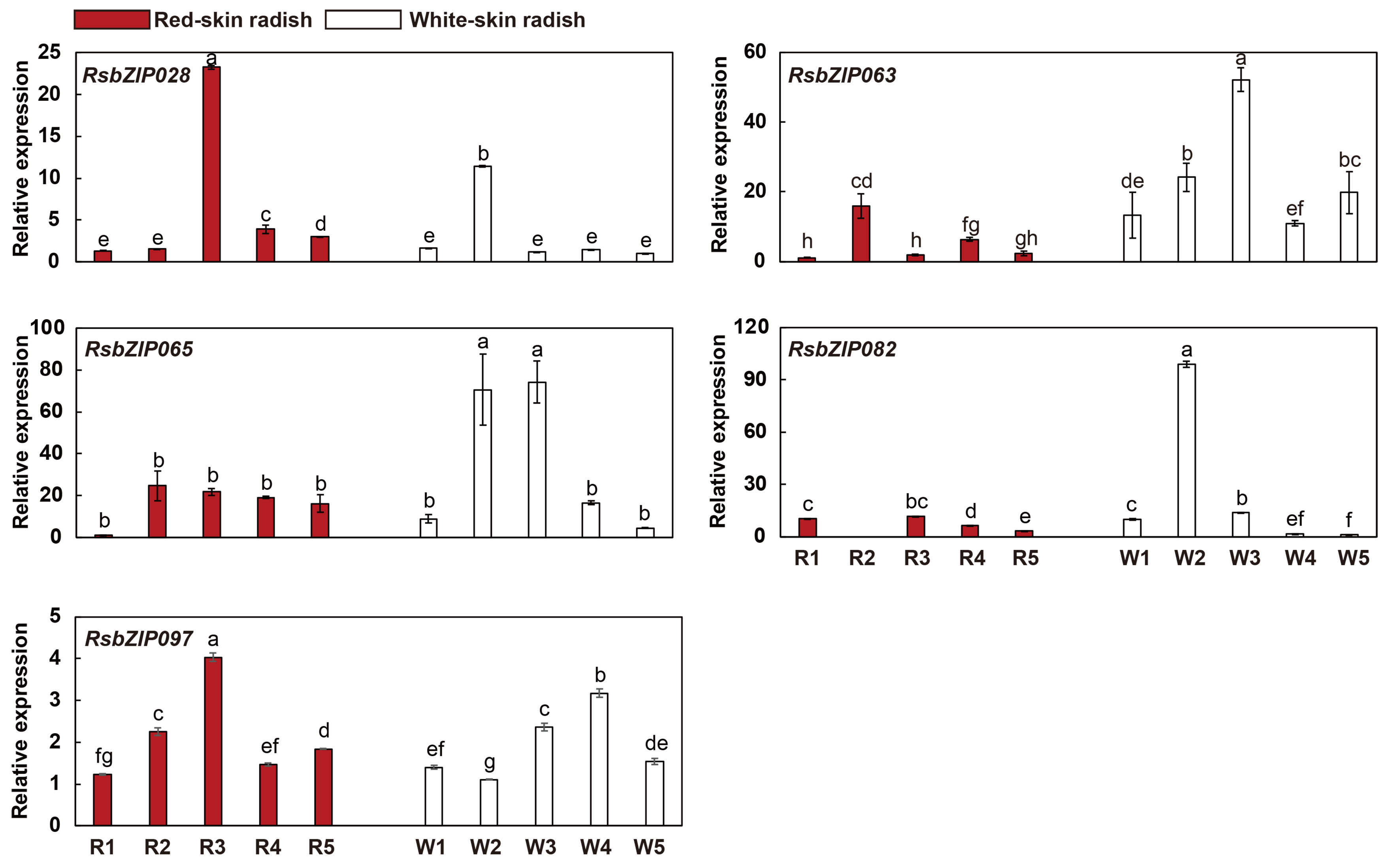


**Figure S4.** Expression profiles of several *RsbZIP* genes from Group H (*RsbZIP028*, *063*, *065*, *082* and *097*) in five red-skin (R1, R2, R3, R4, R5) and five white-skin radish genotypes (W1, W2, W3, W4, W5). Error bars indicate standard deviation based on three replicates. Letters represent significant differences at a 0.05 level based on Duncan’s test
