# Supplementary figures and images for: Genome- and Transcriptome-Wide Characterization of bZIP Gene Family Identifies Potential Members Involved in Abiotic Stress Response and Anthocyanin Biosynthesis in Radish (Raphanus sativus L.)
Source: Int J Mol Sci. 2019 Dec 16;20(24):6334. doi: 10.3390/ijms20246334 (PMC6941039; doi:10.3390/ijms20246334)

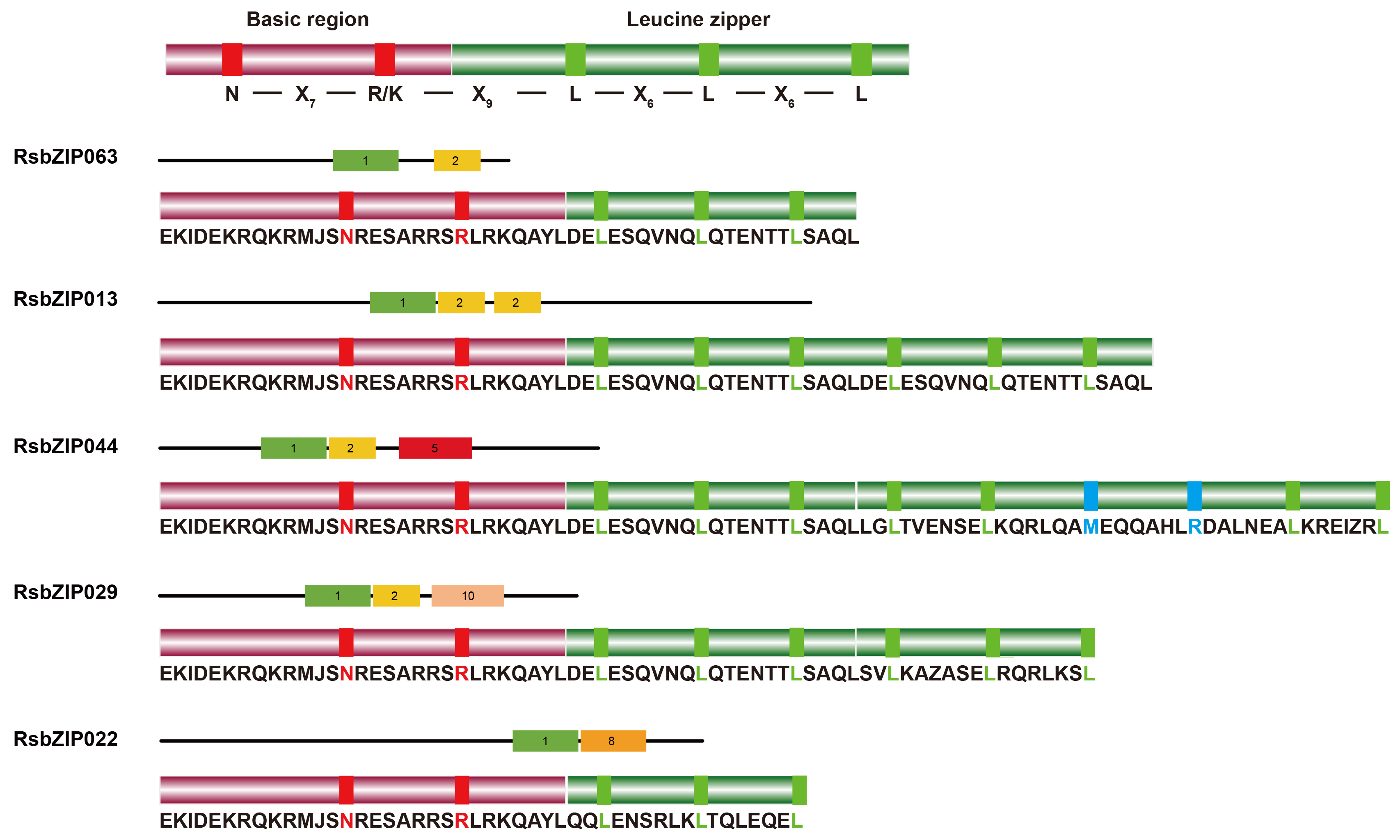


**Figure S1.** The variations of leucine zipper region for RsbZIP proteins

Supplement: Supplementary file 1 [file ijms-20-06334-s001.zip › Figure S1.docx]

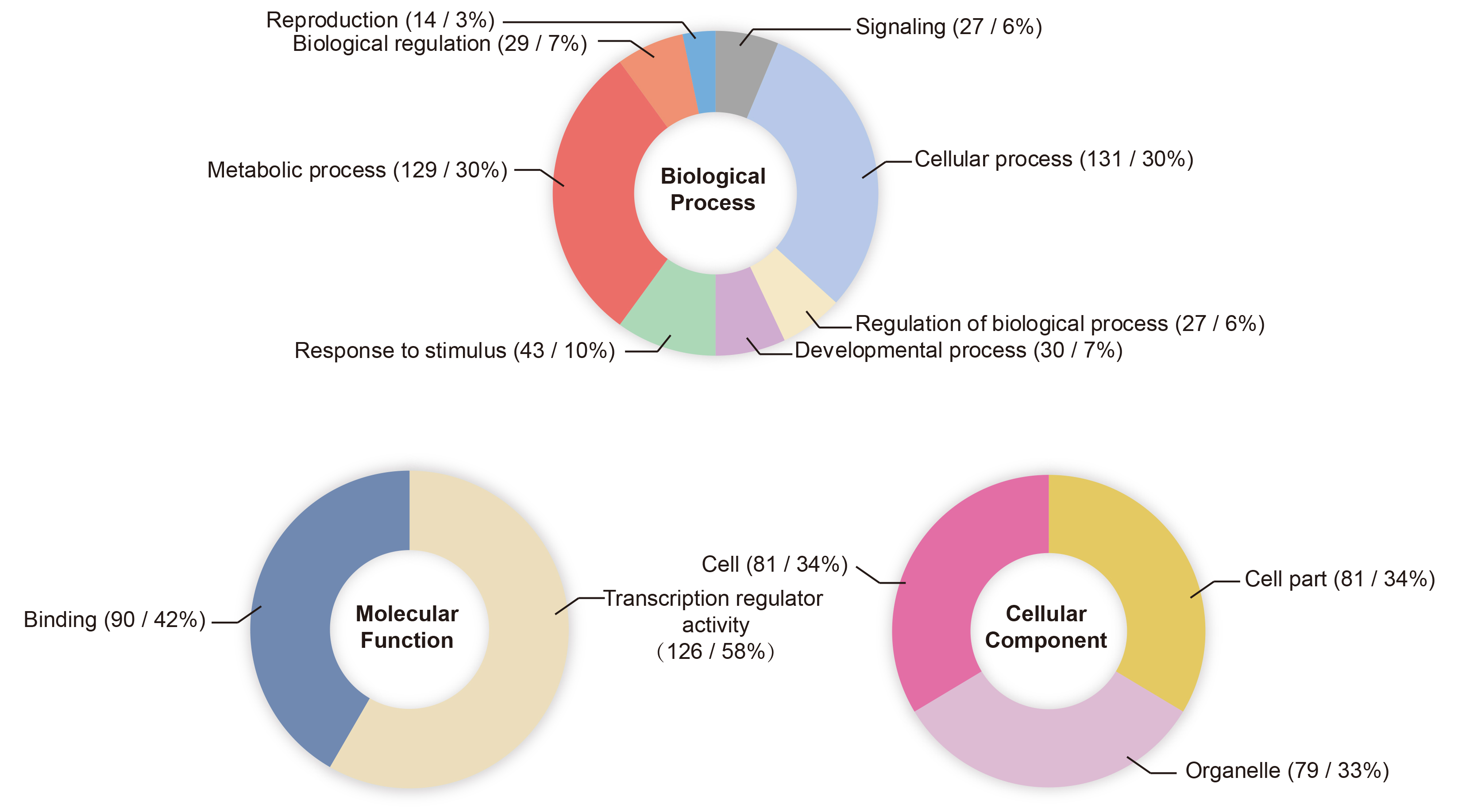


**Figure S5.** GO annotation of RsbZIP proteins

Supplement: Supplementary file 1 [file ijms-20-06334-s001.zip › Figure S5.docx]
